# Enhanced measures of neoantigenicity capture unique tumor-immune interactions across primary melanoma subtypes

**DOI:** 10.1186/s13073-026-01673-3

**Published:** 2026-05-26

**Authors:** Eva R. Shteinman, Grace H. Attrill, Nigel G. Maher, Kiran Gnana, Venkateswar Addala, Yizhe Mao, Umaimainthan Palendira, Jordan W. Conway, Andrew J. Colebatch, Nicola Waddell, Nicholas K. Hayward, John V. Pearson, Georgina V. Long, Richard A. Scolyer, James S. Wilmott, Ismael A. Vergara

**Affiliations:** 1 Melanoma Institute Australia, The University of Sydney, Sydney, NSW Australia; 2 Charles Perkins Centre, The University of Sydney, Sydney, NSW Australia; 3 Faculty of Medicine and Health, The University of Sydney, Sydney, NSW Australia; 4 NSW Health Pathology, Sydney, NSW Australia; 5 Tissue Pathology and Diagnostic Oncology, Royal Prince Alfred Hospital, Sydney, NSW Australia; 6 Cancer Program, QIMR Berghofer Medical Research Institute, Brisbane, QLD Australia; 7 Royal North Shore Hospital, Sydney, NSW Australia; 8 Mater Hospital, Sydney, NSW Australia; 9 School of Biomedical Sciences, University of Queensland, Brisbane, QLD Australia

**Keywords:** Primary melanoma, Cancer genomics, Cancer immunology, Whole-genome sequencing, Neoantigens, Tumor mutational burden, Multiplex immunohistochemistry

## Abstract

**Background:**

As immune checkpoint inhibitor therapies move toward treatment of clinically high-risk localized primary melanoma, there is an increasing need for clear understanding of the tumor-immune interaction across primary melanoma subtypes. Tumor genomic properties play a crucial role in the interaction with the immune system and have been widely explored in metastatic melanoma, but are less understood in the primary context across melanoma subtypes. Persistent tumor mutational burden (pTMB) has been proposed as an enhanced proxy of neoantigenicity in metastatic melanoma compared to standard TMB, but its role in primary disease is not known.

**Methods:**

This study utilised a cohort of 178 predominantly primary melanomas (cutaneous non-acral: *n* = 69, acral: *n* = 36, mucosal: *n* = 73) with available whole-genome sequencing (WGS, *n* = 127) to measure TMB, pTMB, aneuploidy and loss of heterozygosity, as well as multiplexed immunohistochemistry (mIHC, *n* = 140) to identify intratumoral T cells (CD3 + , CD8 + , CD45RO + & CD103 +), B cells (CD20 +), dendritic cells (CD11c +), macrophages (CD68 +) and PD-L1 + cells.

**Results:**

Whilst pTMB is mostly contributed by mutations present in multiple copies across primary melanoma subtypes, distinct families of clonal pTMB capture relationships with CD8 + T cells and CD8 + CD103 + T cells across subtypes not captured by clonal standard TMB. Clonal single-copy pTMB was positively associated with elevated CD8 + T cell (*p =* 0.003) and CD8 + CD103 + T cell infiltration (*p =* 0.008) in cutaneous melanomas. In contrast, clonal multiple-copy pTMB was positively associated with elevated CD8 + CD103 + T cell infiltration in acral (*p =* 0.048) and mucosal (*p =* 0.049) melanomas. Cutaneous primary melanomas had greater proportions of CD8 + T cells (*p =* 0.009) and CD8 + CD103 + T cells (*p =* 0.0002) compared to mucosal melanomas. In turn, CD8 + T cell infiltration was associated with absence of lymphatic invasion (*p =* 0.003), and negatively associated with tumor mitotic rate (*p =* 0.049) in cutaneous melanomas, indicative of less aggressive disease.

**Conclusions:**

Primary melanoma subtypes present significant differences in their immune and genomic landscapes, as well as their interactions. Distinct measures of pTMB carry added value compared to standard TMB for identification of tumour-immune associations across primary melanoma subtypes. Our findings indicate that pTMB is a relevant neoantigenicity marker in primary melanoma with the potential to modulate immune infiltration, leading to more aggressive disease.

**Supplementary Information:**

The online version contains supplementary material available at 10.1186/s13073-026-01673-3.

## Background

Melanoma encompasses a number of subtypes with diverse pathological morphologies, biological characteristics and pathways of progression [1] including cutaneous non-acral melanoma, occurring on hair-bearing skin; acral melanoma, occurring on non-hair-bearing skin of the palms, soles and nail beds; and mucosal melanoma, arising on the mucosal membranes within the body. The genomic landscape of these and other melanoma subtypes are well-documented [2–7].

Tumor mutational burden (TMB), the accumulation of somatic point driver and passenger mutations, has the potential to generate neoantigens that are presented to anti-tumorigenic immune cells via antigen presentation pathways [8], which can in-turn promote immune cell infiltration to the tumour microenvironment. Acquisition of aneuploidy and hyperploidy in tumor cells has been shown to trigger tumor-intrinsic mechanisms that result in immunogenic cell death [9, 10] and conversely, immune evasion [11].

TMB is an FDA-approved biomarker of response to immune checkpoint inhibitor (ICI) treatment in metastatic melanoma, and is generally measured as the total number of non-synonymous mutations per megabase of coding sequence (Mut/Mb). However, this approach to measuring TMB does not always yield reliable associations with response to ICI when melanoma subtypes are stratified [12], or consistently associate with immune infiltration to the tumour [13]. Recently, Niknafs et al*.* [14] have explored a specific family of TMB termed ‘persistent TMB’ (pTMB), which refer to the subset of somatic mutations within a tumor that are located in genomic regions less likely to undergo loss during tumor evolution, including single and multiple copy-regions. pTMB showed a stronger association with both ICI response and an inflamed tumor microenvironment compared to standard TMB across several cancer types, including metastatic melanoma [14]. Other genomic features such as aneuploidy [11], and immune populations such as T cells [15] have also been associated with response to ICI.

As the treatment landscape shifts towards preventative ICI treatment in the adjuvant setting for patients with high-risk primary localized disease [16, 17], a detailed characterisation of immune cell infiltration and its relationship to TMB, pTMB and aneuploidy across primary melanoma subtypes is of interest.

In this study, we utilised multiplex immunohistochemistry (mIHC) and whole-genome sequencing (WGS) to explore the genomic and immune landscape as well as the tumor-immune interaction in a large cohort of primary melanomas spanning cutaneous non-acral, acral and mucosal subtypes. We identify significant differences in the genomic and immune landscape across melanoma subtypes, with distinct measures of persistent TMB [14] capturing associations with the immune landscape that are not captured by the standard approach to measuring TMB. Finally, we identify distinct genomic-immune associations across subtypes that are associated with more aggressive disease as measured by clinical and pathological features within each subtype.

## Methods

### Patients

A total of 178 samples from 177 patients with a diagnosis of primary cutaneous non-acral (hereinafter referred to as cutaneous (*n* = 69)), primary acral (*n* = 36) or mucosal melanoma (*n* = 72) were included in this cohort (Table 1; see Supp. Table S3 for patient level information). All patients were treatment-naïve at the time of specimen collection. Due to the low prevalence of mucosal melanomas across anatomical regions, 26 samples in the mucosal melanoma group with metastatic melanoma tissue available were included for subsequent immune landscape characterisation.

**Table 1:** Summary of melanoma patient clinical and pathological characteristics. For the 26 mucosal metastatic samples, information on pathological factors relating to the primary tumor such as thickness, ulceration status, vascular and lymphatic invasion, TMR and regression are based on the culprit primary tumor. *N* = number of patients; *n* = number of samples

		**Cutaneous (*N* = 69, *n* = 69)**	**Acral (*N* = 36, *n* = 36)**	**Mucosal (*N* = 72, *n* = 73)**
Thickness (mm)		5.7 [1.2–20]	4.85 [1–14.5]	5.2 [0.3–42]
Age at diagnosis		69.6[21.6–91.8]	72.3[52–90.7]	59.4[23.4–90.1]
Sex	Male	45 (65%)	19 (53%)	16(22%)
	Female	24 (35%)	17 (47%)	41(57%)
	Unknown	0 (0%)	0 (0%)	15 (21%)
Sample type	Primary	69 (100%)	36 (100%)	47 (64%)
	Metastatic	0 (0%)	0 (0%)	26 (36%)
Ulceration	Yes	35 (51%)	25 (69%)	43 (60%)
	No	33 (48%)	11 (31%)	4 (6%)
	Unknown	1 (1%)	0 (0%)	25 (34%)
TMR		8 [0–49]	5.5 [1–24]	12 [2–73]
Regression	Absent	22 (32%)	12 (33%)	13(18%)
	Present	42 (61%)	22 (61%)	17 (24%)
	Unknown	5 (7%)	2 (6%)	42 (58%)
Lymphatic invasion	Present	9 (13%)	7 (19%)	10 (14%)
	Absent	49 (71%)	24 (67%)	23 (32%)
	Unknown	11 (16%)	5 (14%)	39 (54%)
Vascular invasion	Present	8 (12%)	6 (17%)	12 (17%)
	Absent	55 (80%)	27 (75%)	26 (36%)
	Unknown	6 (8%)	3 (8%)	34 (47%)
Stage at Diagnosis	I	4 (6%)	4 (11%)	4 (5%)
	II	32 (47%)	14 (39%)	25 (35%)
	III	30 (43%)	15 (42%)	12 (17%)
	IV	2 (3%)	3 (8%)	4 (5%)
	Unknown	1 (1%)	0 (0%)	27 (37%)
Recurrence	Yes	46 (67%)	33 (91%)	50 (69%)
	No	23 (33%)	3 (9%)	4 (6%)
	Unknown	0 (0%)	0 (0%)	18 (25%)
TCGA molecular subtype	BRAF	20 (29%)	2 (6%)	5 (7%)
	NF1	21 (30%)	13 (36%)	18 (25%)
	NRAS	19 (28%)	9 (25%)	6 (8%)
	Triple WT	7 (10%)	11 (30%)	17 (24%)
	Unknown	2 (3%)	1 (3%)	26 (36%)
Experimental info	IHC only	13 (19%)	2 (6%)	36 (49%)
	WGS only	10 (14%)	4 (11%)	16 (22%)
	Both IHC and WGS	46 (67%)	30 (83%)	21 (29%)

Formalin-fixed, paraffin-embedded (FFPE) tissues were utilised for the mIHC staining. Fresh-frozen tumor tissues were used for genomic analyses, as described previously [3]. Prior to use in this study, all patient FFPE samples were sectioned and stained with hematoxylin and eosin to allow for a pathologist to confirm the melanoma diagnosis and histological subtype, as well as to provide additional pathological descriptors.

Among all melanoma patients, 127 had available WGS profiling as part of the Australian Melanoma Genome Project (AMGP) [7] and mIHC was performed on the formalin-fixed, paraffin-embedded (FFPE) tissues in either whole slide tissue sections (*n* = 64) or tumor micro-array (TMA) (*n* = 76). Of these, 135 had available immune profiling that was informative per manual assessment (*ie.* sufficient tumor and no tissue damage). Ninety-two melanoma samples (43 cutaneous, 29 acral and 20 mucosal) had both WGS and immune profiling available.

### WGS, variant calling, purity and ploidy

The WGS data generated in this study is publicly available in European Genome-phenome (EGA) under study accession EGAS00001001552 at https://www.ebi.ac.uk/ega/studies/EGAS00001001552, with dataset accession EGAD00001008798 [7, 18]. Non-synonymous somatic mutations including single nucleotide variants (SNV), dinucleotide variants (DNV), trinucleotide variants (TNV) and small insertion and deletions (indels) were detected from WGS data aligned to GRCh37 human reference as previously described [7]. Briefly, sequence reads were adapter-trimmed using Cutadapt v1.9 [19] and aligned using BWA-MEM v0.7.12 [20]to the GRCh37 assembly. Duplicate reads were marked with Picard MarkDuplicates v1.129 (https://broadinstitute.github.io/picard). The consensus of qSNP v2.0 [21] and GATK HaplotypeCaller v 3.3–0 [22] was used for SNV, DNV and TNV calling. GATK was used to detect indels (1–50 bp). SnpEff [23]was used for variant annotation. Allele-specific copy number calls as well as estimates of purity and ploidy were obtained with ascatNgs [24] and Sequenza version 3.0.0 [25]. For Sequenza, kmi*n* = 500, gamma = 100, normalization.method = median and appropriate sex setting for each sample were used. For each sample, utilization of Sequenza or ascatNgs allele-specific calls, purity and ploidy values were decided based on a set of rules on purity and ploidy distributions across both methods taking into consideration technical and biological factors (see Supplementary Methods).

### Definition of different mutation types

For each non-synonymous somatic mutation, estimates of cancer cell fraction (CCF, defined as the proportion of tumor cells that carry the mutation) and mutant copy number (defined as the number of alleles that carry the mutation) were calculated using the *ccf-annotate-maf* function of the facets-Suite package (https://github.com/mskcc/facets-suite, accessed October 16th, 2023) based on the methodology reported in prior studies [26, 27]. Briefly, mutant copy number was estimated from the observed variant allele frequency after adjustment for tumor purity and local copy number state in tumor cells and normal cells [26]. For CCF, the expected variant allele frequency was modelled as a function of the fraction of tumor cells carrying the mutation, accounting for tumor purity, and local copy number state in tumor cells and normal cells. The likelihood of each possible CCF value in the range 0.1% to 100%, using 0.1% increments, was then estimated from the number of mutant reads and total read depth using a binomial model, generating a posterior distribution across CCF values. The highest-likelihood CCF estimate was selected [27]. The estimates of mutant copy number and CCF were then used to define persistent and clonality status for each mutation (Supp. Table S1). A mutation was regarded as single copy-persistent if the estimated mutant copy number was equal to 1 in regions with a total copy number equal to 1. A mutation was regarded as multiple copy-persistent if the estimated mutant copy number was greater than 1 [14]. A mutation was regarded as persistent if it was either single copy-persistent or multiple copy-persistent. A mutation was regarded as clonal if the 95% confidence interval of the CCF for that mutation intersected with 1, and was regarded as subclonal otherwise [27]. For each sample, the following mutational loads were defined: (i) Non-synonymous mutational load – nsTMB—as the sum of all non-synonymous somatic mutations (also referred to as standard TMB in this work), (ii) Persistent mutational load – pTMB—as the sum of all persistent (single copy and multiple copy) non-synonymous mutations for each sample, (iii) Persistent single copy mutational load – sc-pTMB – as the sum of all persistent single copy non-synonymous mutations, (iv) Persistent multiple copy mutational load – mc-pTMB – as the sum of all persistent multiple copy non-synonymous mutations.

### Aneuploidy and loss of heterozygosity

Total aneuploidy was measured for each sample as the proportion of the segmented autosomal genome that carried either one of the following events in a given genomic region: (i) allelic imbalance, identified in regions with a different major allele copy number and minor allele copy number, (ii) bi-allelic loss, identified in regions with a total copy number of 0, (iii) loss of heterozygosity, identified in regions with a minor allele copy number of 0 and major allele copy number greater than 0, (iv) deletion, identified in regions with either major allele copy number or minor allele copy number equal to 1 in the presence of a WGD event (see Supplementary Methods), (v) amplification, identified in genomic regions with either major or minor allele copy number greater than 1 in the absence of a WGD event, or (vi) amplification, identified in genomic regions with either major or minor allele copy number greater than 2 in the presence of a WGD event. Total loss of heterozygosity was measured for each sample as the proportion of the segmented autosomal genome that had a minor allele copy number equal to 0 and a major allele copy number greater than 0.

### In-silico neoantigen prediction and neoantigenic TMB calculations

HLA class I and class II alleles were predicted from WGS using OptiType v1.3.5 [28] and HLA-HD v1.7.0 [29]. Binding affinity of neoepitopes from somatic point mutations were predicted using pVACseq v4.2.1 [30] with NetMHCpan 4.1 for class I and netMHCIIpan 4.3 for class II [31] and based on VEP v102 annotation [32]. A mutation was deemed neoantigenic if it had one of Best IC50 MHC1 < 500 nM or Best IC50 MHC2 < 500 nM, with Normal VAF < 0.02. Neoantigenic mutational load counterparts for clonal ns-TMB, pTMB, sc-pTMB and mc-pTMB were calculated based on the classification of clonality and persistent status assigned to each mutation.

### Mutational signatures

Individual somatic single nucleotide variants (SNVs) were assigned mutational signatures using SigProfilerExtractor v1.2.1 and SigProfilerAssignment v0.2.3 [32] based on the Cosmic signature reference COSMIC v3.4 [33]. Given the known differences in prevalence of UV mutational processes on acral and mucosal melanomas compared to cutaneous non-acral melanomas, mutational signature assignment in cutaneous non-acral melanomas was done separately from the mucosal and acral counterparts. For each sample, the percentage prevalence of a specific mutational signature was calculated as the number of SNVs assigned to a given signature, divided by the total number of SNVs, multiplied by 100.

### RNA sequencing

The RNA-Seq data used in this study is publicly available in European Genome-phenome (EGA) under study accession EGAS00001001552 at https://www.ebi.ac.uk/ega/studies/EGAS00001001552, with dataset accession EGAD00001008837 [7]. TPM expression values for all genes across samples were calculated as described in Newell et al*.* 2022 [7, 18]. Briefly, RNA-seq reads were trimmed for adapter sequences using Cutadapt v1.11 [19] and aligned using STAR v2.5.2a [34] to the GRCh37 assembly with gene annotation Ensembl release 70. Gene expression was estimated using RSEM v1.2.30 [34] and quality control was carried out using RNA-SeQC v1.1.8 [35]. Low expression genes (TPM < 1 across samples) were excluded. The differentiation signature as published by Hu et al. [36] was calculated for each sample on the TPM values using singscore v1.24.0 with the undirected method [37].

### Multiplexed immunofluorescence staining

mIHC staining was performed on FFPE sections. Both TMA and whole-slide tissue sections were cut at 4-μm and mounted on positively charged SuperFrost Plus slides (Thermo Fisher Scientific). Serial sections were cut for each sample. The slides were left to dry at room temperature overnight, and then stored in a vacuum-sealed cabinet. Prior to staining, the slides were baked at 70 °C for 1 h. Following this, the slides were deparaffinized in xylene and then rehydrated in graded ethanols (100%, 95%, 70%). Slides then underwent heat-induced antigen retrieval (AR) at 95 °C for 20 min in pH 9 AR buffer (Akoya Biosciences, #AR900250ML). After cooling, samples were loaded onto an Autostainer (Dako – Agilent Technologies), and incubated with 3% hydrogen peroxide in TBST for 10 min, followed by sequential staining with either CD8, CD103, PD-L1, CD11c, CD45RO, CD68, CD3, CD20 or SOX10, spread across 3 panels (see Supp. Table S2 for full panel information). The mIHC panels used in this study were based on prior work by Edwards et al. [13]. Slides were incubated with the primary antibodies for 30 min each. Da Vinci Green diluent (Biocare Medical #PD900L) was used to dilute PD-L1, CD8, CD3, CD103, CD45RO, CD20 and SOX10, and 1X Antibody Diluent/Block(#ARD1001EA, Akoya Biosciences) was used to dilute CD11c and CD68. Primary antibodies were then detected using either Mach 3 Polymer HRP (Biocare Medical) for 10 min, or the OPAL Polymer HRP Mouse + Rabbit secondary antibody (Akoya Biosciences, ARH1001EA) for 30 min (Supp. Table S2), and were then visualized using the Opal TSA fluorophores 520, 540, 570, 620, 650, or 690, with a 1:100 dilution in 1 × Plus Automation Amplification Diluent (FP1609, Akoya Biosciences) for 10 min. In between each stain samples underwent antigen retrieval. In the final stain, samples were incubated with Spectral DAPI (1:2000, Akoya Biosciences) diluted in Tris-buffered saline with 0.1% Tween® 20 Detergent (TBST). Appropriate control slides were included in each staining run. Slides were coverslipped with ProLong Diamond Anti-Fade mounting media and allowed to cure overnight in the dark at room temperature before imaging.

### Imaging and expression analysis

All slides were imaged with the Vectra 3.5 Automated Quantitative Pathology Imaging System (Akoya Biosciences), in combination with PhenoChart (Akoya Biosciences) for visualisation, and Inform (version 2.6; Akoya Biosciences) for spectral unmixing using single plex stains as reference slides. 20 × multi-spectral images were acquired and then stitched together to generate single images of the entire tissue for each patient sample. Next, images were imported to HALO (version 3.6, Indica Labs) for quantitative image analysis. Tumor regions were identified in each sample using a machine learning classifier in HALO that was trained on multiple sample images from each cohort and visually inspected prior to use. Only intra-tumoral cells were included in the analysis, and the mIHC expression data was calculated as the percentage of all intra-tumoral cells positive for each respective marker. We included additional measures of T cell proportions in our results: CD8 + and CD3 + CD8- T cells were measured as a proportion of all T cells (CD3 +), and CD103 + CD8 + T cells were calculated as a proportion of total CD8 + T cells. Samples with < 100 tumor cells were excluded from analysis. For immune profiling, three mIHC panels were separately applied (Supp. Table S2), spanning phenotypes for CD3 + CD8-, CD8 + (CD8 + T cells), CD8 + CD103 + tumor resident memory T cells (TRMs), CD45RO + (memory T cells), CD68 + (macrophages), CD11c + (dendritic cells), CD20 + (B cells), PD-L1 + and SOX10 + (melanoma cells). As CD8 was included in the first and third panel, CD8 from the first mIHC panel was used to define CD8 + T cell infiltration, and TRMs (CD8 + CD103 + cells) were also defined based on this panel. CD8- T cells (defined as CD3 + CD8-), and CD8 + and CD8- proportions of overall T cells were measured based on the third mIHC panel, which included CD3 as a marker.

### Statistical analyses

All statistical associations were performed using R Studio (version 4.3.1). Correlation analyses were performed using Spearman’s correlation test. Categorical data was analysed against continuous data using the Mann–Whitney test (for 2 categorical variables) or the Kruskal–Wallis test (for 3 + categorical variables). Associations of genomic and immune features with outcome were assessed using Kaplan–Meier survival analysis with melanoma-specific death as endpoint. Time was measured from Primary Tumor Surgery Date to Date of death or Date of last follow up. Patients with a history of targeted or immune-based therapy were excluded from the analysis. Continuous variables were dichotomized based on median split on all samples with available information. Associations were considered significant if *p <* 0.05. No correction for multiple testing in statistical analyses was pursued due to the exploratory nature of this analysis.

## Results

### Clinical, genomic and immune features of the cohort

Median melanoma thickness of the cohort was 5.2 mm (range 0.3–42 mm.), with no significant differences across subtypes (*p =* 0.43). A significant difference was observed for ulceration status across subtypes (*p <* 0.0001) with acral melanomas presenting with a greater proportion of ulcerated tumours relative to the other histological subtypes (Table 1). Mucosal melanomas had a higher tumor mitotic rate (TMR) relative to the other subtypes (*p =* 0.018). In agreement with the overall prevalence of melanoma subtypes, significant differences were observed for sex (*p =* 0.0001), with cutaneous disease patients being predominantly male [38], and mucosal melanoma patients being predominantly female [39]. In our cohort, patients with acral melanoma were equally distributed across sex, with prior reports indicating different prevalence of males versus females [40–42]. Within each subtype, the proportion of patients with localized primary disease at diagnosis (*ie.* stage I/II disease) was 53% for cutaneous, 50% for acral and 41% for mucosal. Most patients (81%) developed recurrence.

No significant differences were found in immune marker expression between the primary and metastatic mucosal melanomas (Supp. Table S5) and hence were merged for subsequent analysis. TMAs and whole slide sections were combined into a single cohort for data analysis (Supp. Table S6; see Supp. Table S7 and Supp. Results for TMA versus whole slide comparison of immune marker expression).

Genomic features were measured on autosomal chromosomes only, and included total TMB, non-synonymous TMB, persistent non-synonymous TMB from single-copy regions, persistent non-synonymous TMB from multiple-copy regions, extent of loss of heterozygosity (LOH) and extent of aneuploidy (Supp. Table S4—see Methods). Differences in molecular drivers as defined by TCGA [2] across subtypes were in agreement with their known prevalence in the larger population cohort in AMGP [7]. For immune profiling, three mIHC panels were utilized.

### Cutaneous primary melanomas have higher T cell infiltration than acral and mucosal melanomas, whereas acral melanomas have the lowest macrophage infiltration

**Fig. 1:**
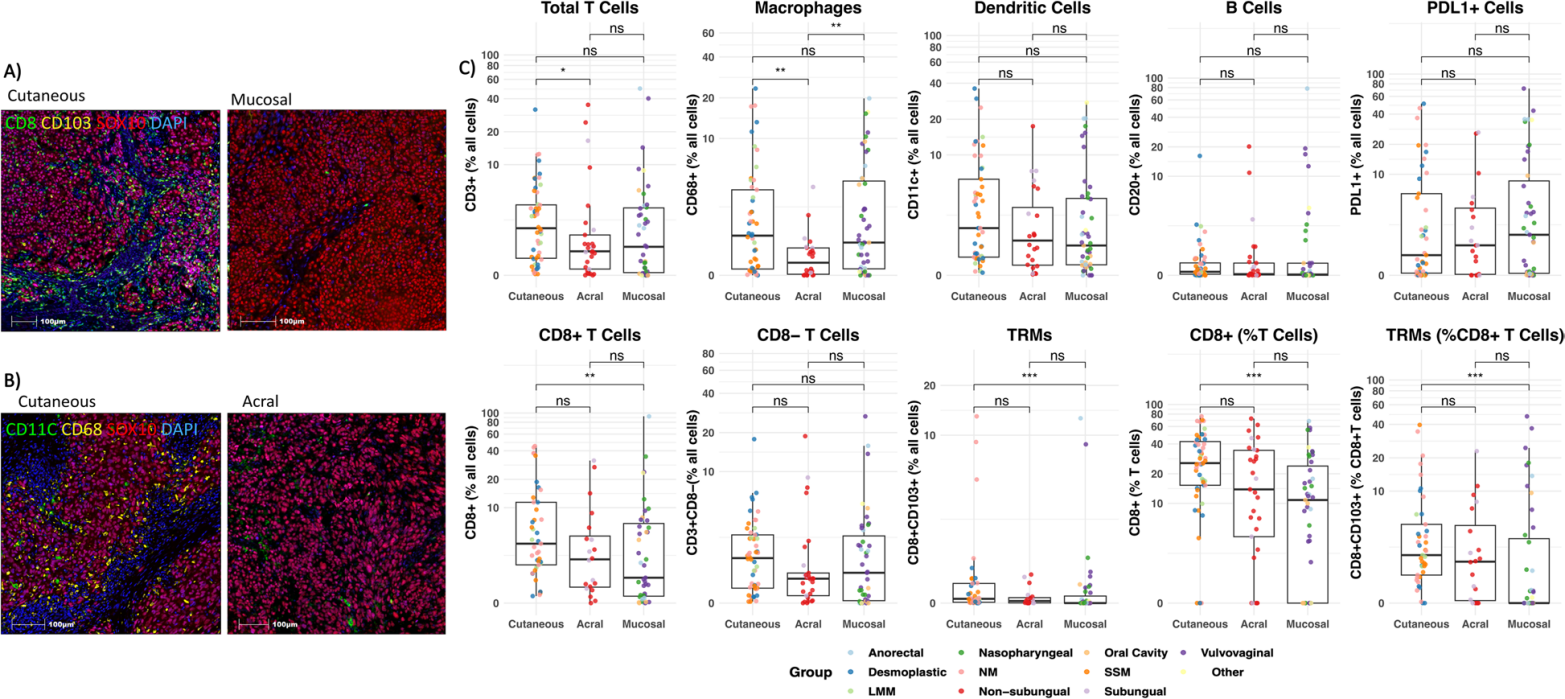
Comparison of immune marker expression across cutaneous (*n* = 56), acral (*n* = 31), and mucosal (*n* = 48) melanomas. **A** Representative mIHC images of cutaneous and mucosal melanoma from the CD8 + CD103 + T cell panel, (**B**) Representative mIHC images of cutaneous and acral melanoma from the macrophage and dendritic cell panel (**C**) Box plots of the proportions of immune cell populations across melanoma subtypes. Datapoints are coloured by clinically relevant group: histological subtype in cutaneous melanomas (desmoplastic melanoma, NM: nodular melanoma, SSM: superficial spreading melanoma, LMM: lentigo maligna melanoma), subungual and non-subungual in acral melanomas and anatomical region (oral cavity, nasopharyngeal, anorectal, vulvovaginal) of the primary tumor in mucosal melanomas. Statistical comparisons are based on the Mann–Whitney test. (ns > 0.05, * ≤ 0.05, ** ≤ 0.01, *** ≤ 0.001, **** ≤ 0.0001). Midline indicates median, whiskers indicate range, box indicates interquartile range

The expression of certain immune markers were found to be higher in cutaneous compared to acral or mucosal melanoma subtypes (Fig. 1; Supp. Table S8). When cutaneous and mucosal melanomas were compared (Fig. 1A), cutaneous melanomas had significantly higher proportions of CD8 + T cells when measured as a proportion of all cells (media*n* = 4% for cutaneous, 1.3% for mucosal, *p =* 0.009, Fig. 1C) and as a proportion of T cells (media*n* = 25% for cutaneous, 11% for mucosal, *p =* 0.0002). Cutaneous melanomas also had a greater proportion of CD8 + Tissue Resident Memory T cells (TRMs) relative to mucosal melanomas when measured as a proportion of all cells (media*n* = 0.12% for cutaneous, 0% for mucosal, *p =* 0.0002) and as a proportion of CD8 + T cells (media*n* = 2.3% for cutaneous, 0% for mucosal, *p =* 0.0003).

Acral melanomas had significantly lower levels of T cells relative to cutaneous melanomas (acral media*n* = 1.05%, cutaneous media*n* = 2.3%, *p =* 0.03), and although not significant there was a trend towards lower levels of TRMs (*p =* 0.07). When CD8 + T cells were measured as a proportion of T cells, acral melanomas trended towards lower proportions relative to cutaneous (acral media*n* = 0.06% l, cutaneous media*n* = 0.17%, *p =* 0.05). Additionally, the proportion of macrophages was lowest in acral melanoma compared to both cutaneous (*p =* 0.002; Fig. 1B) and mucosal (*p =* 0.005) melanomas (media*n* = 0.4% for acral, 1.4% for cutaneous and 1.2% for mucosal, Fig. 1C). No significant differences in CD8- T cells, B cells, PDL1 + cells and CD45RO + memory T cells were observed across subtypes (Fig. 1C, Supp. Table S8). These results indicate an elevated T cell infiltration in cutaneous disease compared to acral and mucosal counterparts, and a lower proportion of macrophage infiltration in acral disease.

Differences in the prevalence of intratumoral immune populations were also observed within primary melanoma subtypes of cutaneous (across histological subtypes), acral (subungual vs non-subungual) and mucosal origin (across primary tumor body region), albeit statistical significance was impacted by the low prevalence within each subtype (See Supp. Results and Supp. Table S9a & Supp. Table S9b). These include a greater proportion of CD8 + T cells in nodular melanomas (NM; *n* = 20) compared to superficial-spreading melanomas (SSM; *n* = 16) (*p =* 0.03) and greater proportion of CD8- T cells in SSM compared to NM (*p =* 0.03) in cutaneous melanomas, a greater proportion of macrophages (*p =* 0.04) and a trend of dendritic cells (*p =* 0.09) in subungual (*n* = 6) compared to non-subungual (*n* = 25) acral melanomas, and a greater proportion of dendritic cells in nasopharyngeal (*n* = 10) compared to oral cavity (n = 6) mucosal melanomas (*p =* 0.04) (Supp. Results). These findings suggest that, in addition to the significant differences in immune infiltration observed between subtypes, immune heterogeneity based on anatomy and histomorphology also exists within subtypes.

### Persistent tumor mutational burden (TMB) is mostly contributed by mutations present in multiple copies across primary melanoma subtypes

Recently, new metrics of TMB have been proposed that focus on *persistent* non-synonymous tumor mutational burden (pTMB) with the potential to encode a neoantigenic load that cannot be easily deleted by the tumor [14]. This can be divided into two subtypes: single-copy pTMB (sc-pTMB) occurring in single-copy genomic regions, and multiple-copy pTMB (mc-pTMB) occurring in multiple copies. The sum of sc-pTMB and mc-pTMB provides total pTMB for a given tumor. The potential heterogeneity in immune populations observed both between and within melanoma subtypes suggests that distinct tumor mutational events across subtypes may drive these differences. To understand the prevalence of standard TMB—defined as the sum of all non-synonymous mutations in each tumor—and pTMB across melanoma subtypes and their impact on immune infiltration, the set of 127 melanomas in this cohort with available WGS spanning primary cutaneous (*n* = 56), primary acral (*n* = 34) and mucosal (*n* = 37) melanomas were assessed (Supp. Table S4). As by definition the extent of pTMB in the tumor is impacted by the extent of aneuploidy (including loss of heterozygosity (LOH)), the percentage of autosomal aneuploidy and autosomal LOH along with ploidy and whole-genome doubling (WGD) were also included in the analysis.

**Fig. 2:**
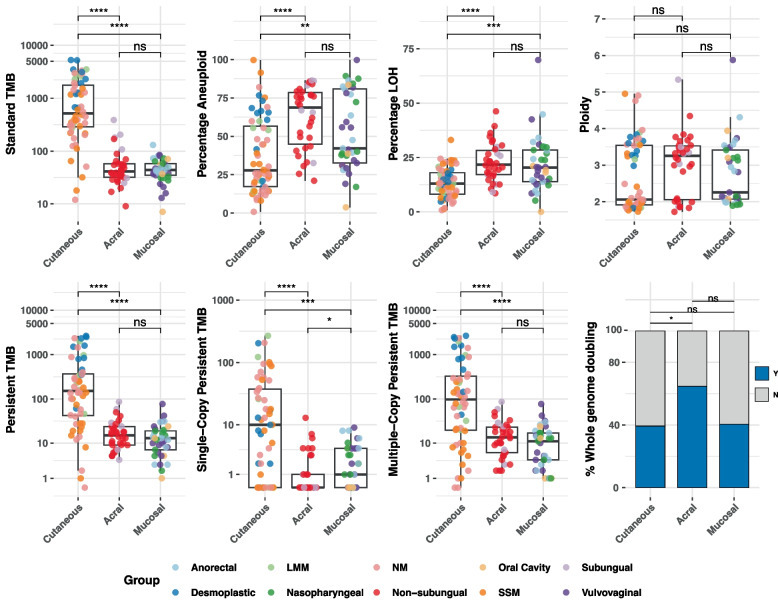
Comparison of the genomic landscape across primary melanoma subtypes. Datapoints are coloured by clinically relevant group: histological subtype in cutaneous melanomas (desmoplastic melanoma, NM: nodular melanoma, SSM: superficial spreading melanoma, LMM: lentigo maligna melanoma), subungual and non-subungual in acral melanomas and anatomical region (oral cavity, nasopharyngeal, anorectal, vulvovaginal) of the primary tumor in mucosal melanomas. Pairwise comparisons were assessed with the Wilcox test for continuous features and the Chi^2^ test for whole-genome doubling. (ns > 0.05, * ≤ 0.05, ** ≤ 0.01, *** ≤ 0.001, **** ≤ 0.0001). Midline indicates median, whiskers indicate range, box indicates interquartile range. LOH = loss of heterozygosity, TMB = tumor mutational burden

As expected, cutaneous primary melanomas carried a significantly higher standard TMB (media*n* = 520.5 mutations) relative to acral (media*n* = 41.5 mutations, *p <* 0.0001) and mucosal (media*n* = 44 mutations, *p <* 0.0001) melanomas, as well as a higher pTMB (media*n* = 151.5 mutations) relative to acral (media*n* = 15 mutations, *p <* 0.0001) and mucosal (media*n* = 13 mutations, *p <* 0.0001) melanomas (Fig. 2), which is in agreement with the known higher exposure to UV damage in the cutaneous group [3]. In all subtypes, the contribution to pTMB was mainly from mutations occurring in multiple copies (media*n* = 98 multiple-copy persistent mutations in cutaneous, 13.5 in acral and 11 in mucosal) compared to mutations occurring in single-copy regions (median 10 single-copy persistent mutations in cutaneous, 0 in acral and 1 in mucosal) (Fig. 2). Acral melanomas showed significantly lower sc-pTMB than mucosal melanomas (*p =* 0.049). While standard TMB and total pTMB were significantly correlated in acral (ρ = 0.7, *p <* 0.0001), cutaneous (ρ = 0.8, *p <* 0.0001) and mucosal melanomas (ρ = 0.5, *p =* 0.0017), inspection of their distribution shows significant variability in the extent of pTMB within the same level of standard TMB (Supp. Figure S1). For example, across samples with 40–60 non-synonymous mutations, there were samples with as little as 2 persistent mutations and samples with as many as 41 persistent mutations.

Differences in standard TMB and pTMB were also observed within subtypes (Supp. Figure S2). In agreement with prior studies [43], desmoplastic melanoma had a significantly higher standard TMB compared to nodular melanoma (NM) (*p =* 0.002) and superficial spreading melanoma (SSM) (*p <* 0.0001), but not lentigo maligna melanoma (LMM) (*p =* 0.6) (Supp. Figure S2). The same pattern of differences was observed for pTMB, driven by differences in mc-pTMB between the desmoplastic group and others (for NM: *p =* 0.001; for SSM: *p <* 0.0001, for LMM: *p =* 0.1)(Supp. Figure S2). No differences in sc-pTMB were observed within cutaneous melanomas. No differences in pTMB within acral and mucosal subtypes were observed.

Significantly higher levels of aneuploidy were observed in acral and mucosal melanomas relative to cutaneous melanomas (median cutaneous = 28% vs. 68% for acral, *p <* 0.0001, and 42% for mucosal, *p =* 0.0016, Fig. 2), in agreement with prior studies [3, 7], Similarly, relative to cutaneous melanomas, both acral and mucosal melanomas presented with higher levels of LOH (median cutaneous = 13%, vs. 22% for acral, *p <* 0.0001, and 20% for mucosal, *p =* 0.0002, Fig. 2). In agreement with prior studies [7,44] a greater proportion of acral melanomas presented with WGD (65%), relative to cutaneous melanomas (39%; chi^2^ *p =* 0.03). There was a trend towards a greater proportion of WGD samples in acral melanomas relative to mucosal melanomas (41%; chi^2^ *p =* 0.07). No significant differences were identified between cutaneous and mucosal melanomas (chi^2^ *p =* 1). No significant differences were identified between the subtypes in terms of ploidy, although a trend was identified towards higher ploidy in acral melanomas (media*n* = 3.25), compared to cutaneous (media*n* = 2.06) and mucosal (media*n* = 2.25), that did not reach significance in Wilcoxon comparisons (cutaneous vs. acral *p =* 0.09; mucosal vs. acral *p =* 0.29; cutaneous vs. mucosal *p =* 0.17).

Overall, these results confirm differences in the extent of standard TMB, aneuploidy and LOH between and within melanoma subtypes and show these differences remain when focusing on distinct families of persistent TMB thar have been proposed as enhanced proxies of neoantigenic load. Additionally, a higher prevalence of mc-pTMB over sc-pTMB is observed between and within subtypes.

Across subtypes, subclonal non-synonymous mutations correspond to a minority of the standard mutational load present in a sample (median proportion of subclonal mutations in cutaneous, acral and mucosal melanomas is 0.0161, 0.0563 and 0.0455 respectively), with these proportions being significantly higher in acral (*p =* 0.02) and mucosal melanomas (*p =* 0.0097) relative to cutaneous melanomas.

The clonality of mutations that encode neoantigens have been deemed clinically and biologically relevant in melanoma and other cancers [45, 46]. However, low purity can reduce sensitivity to detect variants present at lower allele fraction during sequencing [47], thereby biasing the observed subclonal mutational loads. Based on this, we sought to assess whether tumor content influenced the detection of mutational events present only in a fraction of cancer cells by exploring the correlation between purity and the prevalence of mutations deemed subclonal. In agreement with a scenario of lower purity leading to decreased sensitivity during sequencing, subclonal mutations were significantly correlated with purity (ρ = 0.47, *p <* 0.0001), whereas no significant correlation was identified with clonal mutational load (ρ = −0.07, *p =* 0.5; Supp. Figure S3). No associations between purity and melanoma subtypes (Kruskal Wallis p-value = 0.3) or copy number-based genomic features that could indicate biases during sequencing (Supp. Table S10) were identified. Association between purity estimates with %SOX10 + expression in whole slides across the three mIHC panels (Supp. Table S6) identified a significant positive correlation between purity and %SOX10 + expression (panel 1: ρ = 0.32, *p =* 0.02; panel 2: ρ = 0.37, *p =* 0.003); panel 3: ρ = 0.54, *p <* 0.0001), supporting the validity of the purity estimates.

To reduce potential confounding due to purity-dependent detection of subclonal mutations—and given the overall low prevalence of subclonal mutations in our cohort, coupled with the putative lesser role of subclonal mutational load in neoantigenicity- we limit our focus to clonal mutational events for subsequent analyses on the tumor-immune interaction.

### Distinct types of clonal persistent TMB capture relationships with CD8 + T cells and TRMs across subtypes that are not captured by clonal standard TMB

The differences in genomic and immune features between and within melanoma subtypes suggest that distinct tumor-intrinsic events may play a role in modulating the immune context across subtypes. To unveil associations between genomic features and the immune cell context indicative of tumor-immune interactions across melanoma subtypes, the 92 melanomas (43 cutaneous, 29 acral and 20 mucosal melanomas) with available WGS and mIHC were assessed.

First, the association between each clonal measure of TMB: standard TMB, pTMB, sc-pTMB, and mc-pTMB, with each immune population was explored. We found that distinct measures of clonal pTMB showed significant associations with immune populations that were not associated with standard clonal TMB.

**Fig. 3:**
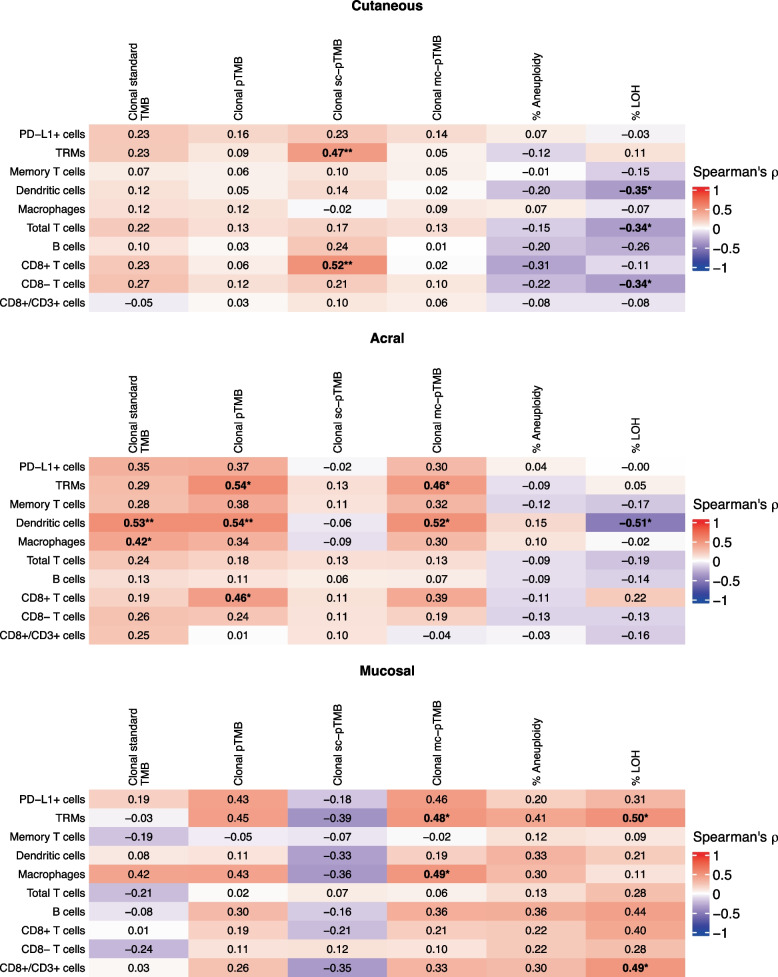
Heatmap demonstrating the associations between different measures of clonal TMB, LOH and aneuploidy with immune cell populations across melanoma subtypes. Cutaneous n = 43, acral n = 29, mucosal n = 20. Spearman’s correlation coefficients (ρ) are shown within cells. (ns > 0.05, * ≤ 0.05, ** ≤ 0.01, *** ≤ 0.001, **** ≤ 0.0001). TMB = tumor mutational burden, sc = single copy, mc = multiple copy, LOH = loss of heterozygosity

In cutaneous melanomas, clonal standard TMB was not significantly associated with any immune population (Fig. 3), whereas clonal sc-pTMB was significantly and positively associated with the proportion of TRMs (ρ = 0.47, *p =* 0.008) and CD8 + T cells (ρ = 0.52, *p =* 0.003). In acral melanomas, clonal standard TMB was significantly positively associated with dendritic cell infiltration (ρ = 0.53, *p =* 0.007) and macrophages (ρ = 0.42, *p =* 0.04), whereas clonal pTMB was significantly associated with TRMs (ρ = 0.54, *p =* 0.02), dendritic cells (ρ = 0.54, *p =* 0.007) and CD8 + T cells (ρ = 0.46, *p =* 0.05) (Fig. 3). Clonal mc-pTMB was also significantly associated with TRMs (ρ = 0.46, *p =* 0.05) and dendritic cells (ρ = 0.52, *p =* 0.01) in this subtype. In mucosal melanomas, clonal mc-pTMB was significantly associated with macrophages (ρ = 0.49, *p =* 0.03) and TRMs (ρ = 0.48, *p =* 0.049) (Fig. 3). Except for macrophages in acral subtype, clonal standard TMB was not associated with an immune population that was not also associated with at least one measure of pTMB (Fig. 3). To assess the impact of mutations deemed neoantigenic on these associations (Supp. Table S1), we generated neoantigen-based versions of the mutational load metrics tested (clonal ns-TMB, pTMB, sc-pTMB and mc-pTMB), and associated these measures with immune cell proportions (Supp. Figure S4, Supp. Table S11). We find that the associations between immune cell proportions and the neoantigenic counterparts of the different TMB metrics remain similar to the above findings, with neoantigen-based clonal pTMB showing a stronger relationship with the immune landscape relative to neoantigen-based clonal nsTMB across subtypes (Supp. Figure S4). Taken together, these results indicate that measures of clonal pTMB are more frequently tied to immune infiltration across cutaneous, acral and mucosal melanomas compared to the standard measure of TMB. Of note, the strength of the associations observed between genomic features and immune populations in acral and mucosal melanomas is likely impacted by the lower prevalence of these rare disease subtypes with mIHC, WGS and comprehensive clinical-pathological information as available in this cohort.

Since the presence of LOH and aneuploidy by definition impact the prevalence of pTMB, we also assessed their association with the immune landscape (Fig. 3). While no associations were observed between the extent of aneuploidy and immune cell populations across all melanoma subtypes, distinct associations between LOH and immune cell populations were identified. In cutaneous melanoma, the extent of LOH was negatively correlated with proportion of dendritic cells (ρ = −0.35, *p =* 0.03), proportions of CD8- T cells (ρ = −0.34, *p =* 0.03) and total T cells (ρ = −0.34, *p =* 0.03). In acral melanomas, LOH was negatively correlated with proportion of dendritic cells (ρ = −0.51, *p =* 0.01). In contrast, in mucosal melanomas, the extent of LOH was positively associated with proportion of TRMs (ρ = 0.5, *p =* 0.04) and CD8 + T cell proportions (of total T cells, ρ = 0.49, *p =* 0.04). Mucosal melanomas with a WGD event had higher proportions of TRMs relative to mucosal melanomas without WGD (WGD median TRM proportio*n* = 0.36%, non-WGD median TRM proportio*n* = 0%, *p =* 0.049), as well as macrophages (WGD median macrophage proportio*n* = 2.74%, non-WGD median macrophage proportio*n* = 0.71%, *p =* 0.048). No further associations with WGD status across subtypes were identified and no significant associations of ploidy with immune cell populations were identified (Supp. Table S11). These findings suggest that LOH and WGD have a tissue-specific impact on the immune landscape.

Additional genomic features yielded limited associations with the immune context. Mutational signature analysis (see Methods) revealed a trend of positive association between UV signature and CD8 + T-cell proportions in cutaneous melanomas that did not reach statistical significance (r = 0.34; *p =* 0.054). No other associations between mutational signatures and immune populations were identified across subtypes (Supp. Table S4, Supp. Table S11). Assessment of TCGA-based driver mutation status against immune features -restricted to cutaneous non-acral melanomas due to prevalence of mutational events- showed no associations with immune features (Supp. Table S11).

Additionally, the de-differentiation phenotypes of melanoma have been associated with proliferation, invasion and resistance for both targeted and immune checkpoint inhibitor therapies [48–50]. To explore the relationship between melanoma differentiation status and the immune landscape, we assessed genes associated with melanoma differentiation status, as defined by Hu et al*.* [36] in a subset of patients in our cohort with available RNA-seq and immune profiling data (*n* = 17 acral, *n* = 8 cutaneous, *n* = 11 mucosal, Supp. Table S6). In acral melanomas, the differentiated gene signature was negatively correlated with the proportion of macrophages (ρ = −0.54, *p =* 0.03). In mucosal melanomas, this signature was negatively correlated with CD3⁺CD8⁻ cell proportions (ρ = −0.73, *p =* 0.04). In contrast, no significant correlations between the differentiated gene signature and immune markers were observed in cutaneous melanomas. Whilst this lack of association could be due to small number of cases with available RNA-Seq and immune profiling information in the cutaneous subtype, given the divergent immunogenicity across the melanoma subtypes, the immunogenic cutaneous melanoma subtype may have multiple redundant mechanisms beyond differentiation to enable immune escape.

### The associations between pTMB and immune cell populations are linked to more invasive and proliferative disease

**Fig. 4:**
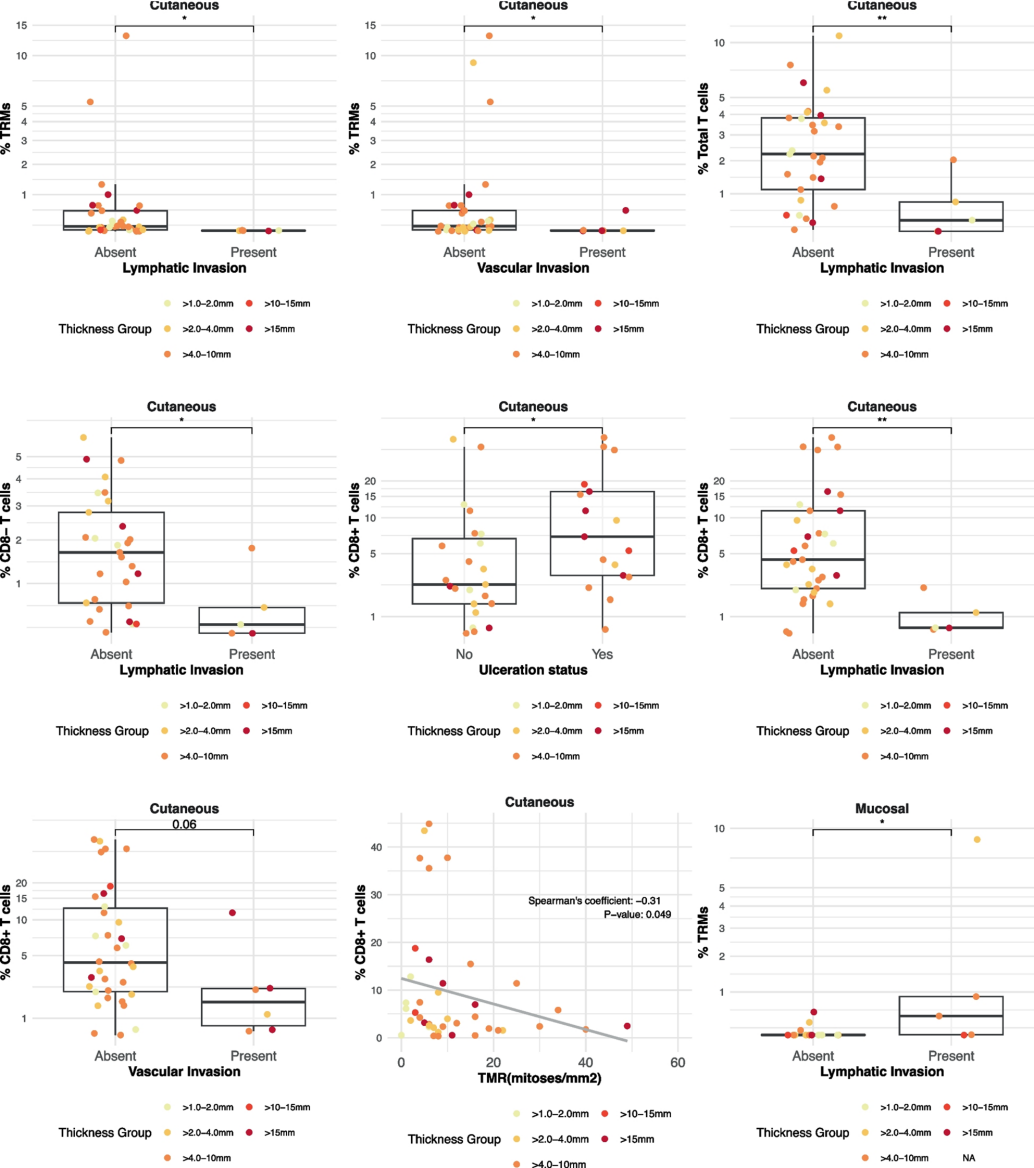
Associations between the immune landscape and pathological factors in primary cutaneous and mucosal melanomas. Immune features were included based on their significant associations with pTMB, LOH and WGD. Individual data points are coloured according to Breslow thickness grouping. Pairwise comparisons were assessed with the Wilcox test. Correlations were assessed via scatter plot with Spearman’s correlation test. (ns > 0.05, * ≤ 0.05, ** ≤ 0.01, *** ≤ 0.001, **** ≤ 0.0001). (TMR = Tumor Mitotic Rate). Midline indicates median, whiskers indicate range, box indicates interquartile range

To understand if the identified associations between pTMB, LOH and WGD with immune context have the potential to lead to more invasive and proliferative disease, the associations of the impacted immune cell populations with pathological factors were assessed (Fig. 4).

In cutaneous melanomas, immune cell populations impacted by pTMB and LOH were associated with less aggressive features of disease. Specifically, CD8 + T cell proportion and TRM (positively associated with clonal sc-pTMB) as well as Total T cell and CD8- T cell proportions (negatively associated with LOH) were associated with absence of lymphatic invasion (*p =* 0.003 for CD8 + T cells, *p =* 0.01 for TRM, *p =* 0.007 for Total T cells, *p =* 0.009 for CD8- T cells, Fig. 4). Higher TRMs were also associated with absence of vascular invasion (*p =* 0.04, Fig. 4). CD8 + T cell proportions had a borderline association with less proliferative disease as measured by tumor mitotic rate (TMR) (ρ = −0.3, p- = 0.05) and a trend with absence of vascular invasion (*p =* 0.06) (Fig. 4). At the same time, CD8 + T cell proportions were associated with higher rates of ulceration in cutaneous melanomas (*p =* 0.03, Fig. 4)—a more aggressive feature of disease—with TRMs showing a positive but not significant trend of association with ulceration (*p =* 0.1). This is in agreement with a pro-inflammatory microenvironment and increased expression of proteins involved in tumor antigen presentation promoted by ulceration [51]. No associations between dendritic cells (negatively associated with LOH) and pathological features were observed in this subtype.

In acral melanomas, the proportion of CD8 + T cells – positively associated with clonal pTMB—the proportion of TRMs – positively associated with clonal pTMB and mc-pTMB– and the proportion of dendritic cells – negatively associated with LOH and positively associated with clonal pTMB and mc-pTMB—were not found to be associated with clinical or pathological features in this subtype.

In mucosal melanomas, TRMs – which were positively associated with clonal mc-pTMB, LOH and WGD—was associated with an increased presence of lymphatic invasion (*p =* 0.02, Fig. 4). No associations between macrophages – positively associated with clonal mc-pTMB and WGD – and CD8 + T cell proportions (of total T cells) – positively associated with LOH – with clinical-pathological factors were identified.

We identified a limited number of associations between pathological factors and immune populations that were not related to genomic features (see Supp. Table S12 for all associations between immune populations and pathological factors), which indicates the presence of an immune response that operates independently of tumor-intrinsic features.

Taken together, these results suggest a scenario of distinct persistent mutational events, LOH and WGD modulating the presence of different immune cell populations, impacting the aggressiveness of primary disease across melanomas subtypes.

### Standard and novel TMB measures are associated with melanoma-specific survival in cutaneous non-acral melanomas

To understand the potential prognostic significance of the genomic and immune features characterised in this study, we measured their association with melanoma-specific survival (MSS) in treatment-naïve acral, cutaneous and acral melanoma patients (see Methods).

In cutaneous non-acral melanomas, higher clonal standard TMB (median split = 516.5 mutations, median survival ‘Low’ grou*p =* 940 days, median survival ‘High’ grou*p =* Not Achieved, *p =* 0.02), clonal neoantigenic TMB (median split = 430.5 mutations, median survival ‘Low’ grou*p =* 940 days, median survival ‘High’ grou*p =* Not Achieved, *p =* 0.02) and clonal pTMB (median split = 137.5 mutations, median survival ‘Low’ grou*p =* 4158 days, median survival ‘High’ grou*p =* Not Achieved, *p =* 0.04) were associated with improved MSS (Supp. Figure S5, Supp. Table S13). Additionally, presence of a *BRAF* driver mutation was associated with worse MSS. These results highlight a role for the extent of clonal neoantigenic burden present in the primary tumor on melanoma-specific death. Association of immune features with MSS revealed that higher memory T cells (CD45RO) were associated with worse MSS (median split 6.5%, *p =* 0.003). No associations were identified in acral or mucosal melanomas.

## Discussion

While substantial research has been dedicated to characterising the genomic and immune landscape of metastatic melanoma, comparatively less attention has been paid to early-stage disease, and especially to rarer subtypes of melanoma such as acral and mucosal melanoma. In this study, we sought to characterize the genomic and immune landscape of a large cohort of primary melanomas (as well as an additional subset of metastatic mucosal melanomas), across cutaneous, acral and mucosal subtypes, to probe the tumor-immune interaction shaping disease trajectories.

We report that pTMB captures relationships with the immune landscape that are not captured by standard TMB measures. Clonal measures of pTMB – but not standard TMB—were associated with TRMs across all three melanoma subtypes, in addition to macrophages in mucosal melanoma, and CD8 + T cells in cutaneous and acral melanoma. Interestingly, rather than one measure of pTMB being consistently tied to immune infiltration across the melanoma subtypes, we found that distinct families of pTMB held relevance for each subtype—sc-pTMB was tied to immune infiltration in cutaneous melanomas, and mc-pTMB in acral and mucosal melanomas. Prior studies by our group have demonstrated that melanoma subtypes vary in their genomic landscape, including the extent of point mutations and copy number alterations [3, 7]. The finding that different pTMB measures are linked to immune infiltration across subtypes likely stems from the significant differences in prevalence of LOH and overall aneuploidy between cutaneous and acral/mucosal melanoma subtypes, which by definition impact the prevalence of pTMB.

Additionally, we report that some immune populations uniquely linked to pTMB (and not standard TMB) were significantly associated with less aggressive disease, as reflected by the increase in CD8 + T cells and TRMs associated with absence of lymphatic and vascular invasion as well as increased CD8 + T cells associated with reduced tumor mitotic rate in cutaneous non-acral melanomas. This indicates that pTMB captures a biological phenomenon that is distinct from non-synonymous TMB – one more tied to neoantigenicity and immune infiltration, manifesting in more contained and less proliferative disease within the tumor. In line with this, Niknafs and colleagues [14] report that high pTMB is associated with higher rates of response to ICIs in patients with metastatic melanoma. Additionally, and in agreement with Niknafs et al*.*, we find that restricting the definitions of TMB and pTMB to mutations deemed neoantigenic based on predicted HLA allele binding affinity does not significantly impact the strength of the associations of these metrics with immune cell populations, likely as a reflection of the known limitations of *in-silico* neoantigen prediction methods to identify neoepitopes that elicit an immune response.

While our study is focused on a primary melanoma cohort that did not receive treatment with immunotherapies, as datasets of patients treated with immunotherapies in the primary localised setting (stage IIB/IIC) become available the associations between pTMB and response to ICI should be performed. Based on the genomic differences across subtypes reported here in the primary setting, as well as prior studies documenting their confounding role when assessing response in the metastatic setting [12], a robust association between pTMB and anti-PD1-based treatment response requires sizeable cohorts that incorporate sufficient events across cutaneous non-acral, acral and mucosal disease. This is further compounded by differential biology of tumours in response to anti-PD1 in the presence versus absence of anti-CTLA4 [52], requiring further stratification by treatment modality. As the melanoma treatment landscape shifts towards preventative ICI treatment for patients with high-risk localized disease at diagnosis [16, 17], our findings and those of others [12, 52] coupled with the results of Niknafs et al. [14] provide a strong rationale for further exploration of this measure of TMB in cohorts of high-risk primary melanomas treated with ICI.

The associations identified between standard and novel clonal measures of mutational burden with MSS in treatment-naïve cutaneous melanoma patients highlight a prognostic role for metrics that capture the extent of neoantigenicity present in the primary tumor. We also identified an association between increased proportions of memory T cells (CD45RO) and worse melanoma-specific survival, although CD45RO memory T-cells are a mixed population of T cells which can be viral-specific and not tumor-specific [53]. While tumor-specific T cells (CD39 +) are generally associated with favourable outcomes in primary melanoma [54], additional tumor-specific TRMs have been linked to improved survival in metastatic melanoma [55] and a range of other cancers [56–61]. However, our data also revealed a significant positive association between CD8 + T cells and the presence of ulceration, as well as a trend with TRMs. Hence, we suspect that ulceration may be a confounding factor in the association between TRMs and survival in our cohort. It has previously been shown that ulcerated primary tumors are more proliferative, have greater vessel invasion and up-regulate pro-inflammatory cytokines [62], causing a generally increased inflammatory state. This is also hypothesised to underly the association between patients with ulcerated primary melanomas and improved response to interferon therapy [63]. Therefore, we suggest that in our cohort, the poor prognosis identified as associated with TRMs may stem more from the increased proliferative and invasive state of ulcerated tumors and its associated inflammation, rather than truly being caused by CD8 + T cells.

We identified distinct associations between the extent of LOH and immune cell populations across the melanoma subtypes. In cutaneous non-acral and acral melanomas, the extent of LOH was negatively correlated with specific immune populations, while in mucosal melanomas LOH positively associated with immune infiltration. Again, we found that the particular immune populations linked to this genomic feature were unique to each subtype. We have shown previously that acquisition of LOH in large genomic regions during progression to lethal melanoma may act as an efficient mechanism of immunoediting neoantigenic load [45]. The unexpected opposite direction in the association between LOH and immune cell populations across subtypes may be explained by the extent to which an LOH event enables immune escape via deletion of neoantigens in the lost allele (*e.g.* in cutaneous non-acral and acral melanomas) versus the extent to which the same LOH event generates persistent neoantigens in the remaining alleles (*e.g*. in mucosal melanomas). Overall, these results suggest that acquisition of LOH events during tumor evolution may have a complex and subtype-specific effect on immune context modulation.

A major theme throughout our results was that the melanoma immune landscape is extremely heterogenous, not only between subtypes but also within subtypes. In particular, we identified several significant differences in the proportion of distinct immune populations between mucosal melanomas of nasopharyngeal, oral cavity and vulvovaginal origin. Findings from genomic studies indicated stark mutational differences between mucosal melanomas from primary regions that are anatomically close together – *e.g.* between the vulva and vagina, or between the nasal cavity and the sinuses [64] – which further compounds difficulties in defining a single definitive set of characteristics for these mucosal subtypes. Despite the differences identified between mucosal tumors of different anatomical location, clinical studies have reported that mucosal primary tumor site is not associated with response to ICI treatment [65–67], which may indicate that distinct biological features of mucosal melanomas based on primary site become less clinically meaningful once the tumor has metastasised. To this end, the lack of significant differences in the immune context of primary and metastatic mucosal melanomas identified here suggest that tumor-intrinsic features acquired in the primary tumor that enable immune modulation may also have the same modulating ability in distant sites. However, it is important to keep in mind that clinical trials typically group mucosal melanomas into categories such as head and neck or naso-oral region, genitourinary tract, and gastrointestinal tract [65, 66], which may be too broad to be truly sensitive to the differences in immunogenicity between primary regions. The rarity of mucosal melanoma poses difficulties in generating cohorts large enough for comprehensive comparisons of the tumor microenvironments between tumors of different primary site, making this an ongoing, unresolved question in the field. Given the generally poor survival outcomes of patients with this disease subtype, this question warrants further exploration.

In the context of ICI treatment, clonal mutational and neoantigen burden have been reported to be superior predictors of response when compared to total mutational and neoantigen burden [46, 68]. Because of this, as well as the potentially confounding relationship identified between subclonal mutational burden and tumor purity, we decided to limit our analyses to clonal mutations only. While the overall proportion of subclonal mutations in all subtypes was low (median < 1%), we did identify significantly higher proportions of subclonal mutations in acral and mucosal melanomas compared to cutaneous melanomas, while cutaneous melanomas presented with a higher proportion of clonal mutations. This difference is unlikely to be due to tumor content since there were no differences in purity distribution across melanoma subtypes. This finding builds on work by Tarantino et al*.*, in which it was also shown that acral and mucosal melanomas present with higher genomic heterogeneity compared to cutaneous melanomas in the metastatic setting [44]. The difference in exposure to ultraviolet radiation, which acts as a potent exogenous mutagen, is thought to underlie the lower mutational heterogeneity of cutaneous melanomas relative to acral and mucosal subtypes [3]. Subclonal mutations are by no means inert, however—subclonal mutations in *PIK3CA* can act as driver mutations and contribute to the progression of metastatic melanoma [69], and cells with less immunogenic mutational subclones may actually expand in response to ICI treatment [68], contributing to a tumor’s ability to evade the immune system. Additionally, the proportion of subclonal mutations has been associated with failure to respond to ICIs [12, 44]. In this sense, the predictive and prognostic power of subclonal standard and new measures of TMB within melanomas may be clarified by longitudinal studies with intra-patient sampling.

This study had several limitations. First, the study was limited by small sample size numbers for some group comparisons, primarily within acral and mucosal subtypes, in virtue of the low prevalence of these melanoma subtypes within the population. Still, the careful stratification by subtype and the strong focus on the primary setting was part of the study design and essential to identify the patterns reported here. Future studies focused on validation cohorts with multi-omics integration and well-annotated clinical and pathological features as reported here, as well as functional assays that assess underlying mechanisms will shed further light on the translational value of our findings. Second, averaging immune cell proportions for each sample enabled appropriate analyses, however the density of each marker within different locations of the tissue was not taken into account. While the benefit of this approach is that it allows for a broad representation of overall infiltration levels of different immune populations, it is important to note that studies have shown that capturing the spatial organisation of immune cells within and in the surrounding regions of a tumor can unveil to a greater extent the functional interactions of cells within melanoma and other cancers [54, 70, 71]. Finally, in our study the analysis was limited to intratumoral regions only, so as to allow for the accurate comparison of TMA samples –which were intratumoral only – with whole slides. Mesenchymal interactions in stromal regions of the tumor play a role in shaping the progression of the tumor [72], and recently, in a cohort of adolescent and young adult melanoma patients, it has been shown that immune populations thought to contribute to resistance to ICIs are localized to the stroma [73]. Future studies across melanoma subtypes that incorporate spatial analysis of both intratumoral and stromal regions into their methodology will be of great benefit to the field.

## Conclusion

In conclusion, this study highlights the significant differences in the immune landscape, genomic landscape, and their interaction across cutaneous, acral and mucosal melanoma subtypes. We report that new measures of TMB [14] capture relationships with critical immune populations that are associated with pathological factors, and are not captured by standard approaches to TMB alone. These findings provide a justification for further assessment of this approach to TMB in additional melanoma cohorts.

## Supplementary Information

Supplementary Material 1.

Supplementary Material 2.

Supplementary Material 3.

Supplementary Material 4.

## Data Availability

The DNA and RNA datasets generated and/or analysed during the current study are available in the European Genome-Phenome repository, https://www.ebi.ac.uk/ega/studies/EGAS00001001552 (18) with data set accessions EGAD00001008798 (WGS) and EGAD00001008837 (RNA-seq). All mIHC data used in this study is available in the supporting files (Additional file 2, Table S6).

## Abbreviations

TRM: Tissue-resident memory cells
WGS: Whole-genome sequencing
FFPE: Formalin-fixed paraffin embedded
TMB: Tumor mutational burden
pTMB: Persistent tumor mutational burden
ns-TMB: Non-synonymous tumor mutational burden
mc-pTMB: Multiple copy persistent tumor mutational burden
sc-pTMB: Single copy tumor mutational burden
WGD: Whole genome doubling
TMA: Tumor micro-array
LOH: Loss of heterozygosity
mIHC: Multiplex Immunohistochemistry
UV: Ultraviolet
