# Supplementary material for: Enhanced measures of neoantigenicity capture unique tumor-immune interactions across primary melanoma subtypes
Source: Genome Med. 2026 May 26;18:106. doi: 10.1186/s13073-026-01673-3 (PMC13390141; doi:10.1186/s13073-026-01673-3)
Supplement: Supplementary file 1 — Supplementary Material 1. [file 13073_2026_1673_MOESM1_ESM.docx]

**Supplementary figures**

**Figure S1. Correlation plot showing the relationship between standard TMB and pTMB across melanoma subtypes.** Datapoints are coloured by melanoma subtype. For the X and Y axis, a pseudo-logarithmic scale was used.

**Figure S2. Comparison of different measures of TMB across primary melanoma subtypes of cutaneous, acral and mucosal.** Datapoints are coloured by clinically relevant group: histological subtype in cutaneous melanomas (desmoplastic melanoma, NM: nodular melanoma, SSM: superficial spreading melanoma, LMM: lentigo maligna melanoma), subungual and non-subungual in acral melanomas and anatomical region (oral cavity, nasopharyngeal, anorectal, vulvovaginal) of the primary tumor in mucosal melanomas. Statistical comparisons are based on the Mann-Whitney test. (ns >0.05, * ≤ 0.05, ** ≤ 0.01, ***≤ 0.001, ******** ≤ 0.0001).

**Figure S3.** **Correlation plots comparing the association between clonal and subclonal ns-TMB with sample tumor purity in primary cutaneous melanomas.** Statistical analysis based on the Spearman’s correlation test. For the Y axis, a pseudo-logarithmic scale was used. The grey band represents the LOESS curve for each correlation.

**Figure S4. Heatmap demonstrating the associations between different neoantigenic-based measures of TMB with immune cell populations across melanoma subtypes.** Cutaneous n = 43, acral n = 29, mucosal n = 20. Spearman’s correlation coefficients (ρ) are shown within cells. (ns >0.05, * ≤ 0.05, ** ≤ 0.01, ***≤ 0.001, **** ≤ 0.0001). TMB = tumor mutational burden, sc = single copy, mc = multiple copy, pTMB = persistent TMB, neo = neoantigenic.

**Figure S5. Kaplan-Meier curves for features significantly associated with melanoma-specific death.** Time variable is represented in days (X axis). Survival probability corresponds to melanoma-specific survival (Y axis).
