# Supplementary material for: Enhanced measures of neoantigenicity capture unique tumor-immune interactions across primary melanoma subtypes
Source: Genome Med. 2026 May 26;18:106. doi: 10.1186/s13073-026-01673-3 (PMC13390141; doi:10.1186/s13073-026-01673-3)
Supplement: Supplementary file 3 — Supplementary Material 3. [file 13073_2026_1673_MOESM3_ESM.docx]

*Supplementary Results*

1. TMA v whole slide sections

TMA and whole slide sections were compared in terms of their immune marker expression prior to being merged into a single cohort. No significant differences were identified between TMAs and whole slides in the cutaneous and acral samples (Supp. Table 7), however significant differences were identified in the mucosal samples for the markers CD11C, CD20, CD45RO and CD3, with higher immune infiltration found in the mucosal whole slides. Pathological review of these particular cases confirmed they have higher TILs when compared to the mucosal samples included in TMAs, and we concluded that these findings reflected legitimate sample-based differences between the cases.

1. Intra-subtype results

The heterogeneity between melanoma subtypes present in this cohort prompted the assessment of differences within cutaneous, acral and mucosal melanomas. Across the 56 cutaneous melanoma patients with available mIHC information, superficial spreading (SSM n=16), nodular (NM n=20) and desmoplastic melanomas (n=14) are the most prevalent, with also lentigo malignant (LMM n=6) present. Acral melanomas include subungual - defined as melanomas of the nail apparatus (n=6) - and non-subungual – defined as melanomas of the palms and soles (n=25). Mucosal melanomas were classified based on the anatomical region of the primary tumour, into vulvovaginal (n=22), oral cavity (n=6), nasopharyngeal (n=10), anorectal (n=8) and conjunctival (n=1). 1 patient with primary mucosal melanoma did not have available classification.

Within subtypes, significant differences were observed in the composition of intratumoural immune profile (Supp. Table 9a & 9b). Within cutaneous primary melanomas, NMs presented with a greater level of CD8+ T cells as a proportion of T cells relative to SSMs (NM = 36%, SSM = 19%, p = 0.027). Additionally, NMs presented with a trend towards greater proportions of PD-L1+ cells (NM = 1.9%, SSM = 0.4%, p = 0.059). In contrast, SSMs presented with greater proportions of CD4+ T cells, as a proportion of T cells, relative to NMs (NM = 64%, SSM = 81% p = 0.027).

Within acral melanomas, subungual melanomas presented with a significantly greater proportion of macrophages (1% for subungual, 0.17% for non-subungual, p = 0.04), and trended towards a greater proportion of dendritic cells (6% for subungual, 1.1% for non-subungual, p = 0.08), relative to non-subungual melanomas.

Within mucosal melanomas, nasopharyngeal samples presented with greater immune infiltration relative to oral cavity samples. Specifically, we identified greater proportions of dendritic cells (1.18% for nasopharyngeal vs. 0.46% for oral cavity, p = 0.05), greater T cell proportions of CD8+ T cells (median = 0% for oral cavity vs. 15% for nasopharyngeal, p = 0.04), and a trend with PD-L1+ cells (0.073% for oral cavity vs. 2.27% for nasopharyngeal, p = 0.07). Patients with vulvovaginal samples trended towards greater immune infiltration relative to oral cavity samples, including memory T cells (10.38% for vulvovaginal vs. 2.44% for oral cavity, p = 0.08), and T cell proportions of CD8+ T cells (11.5% for vulvovaginal, 0% for oral cavity, p = 0.09) These findings suggest that, in addition to the large differences in immune infiltration observed between subtypes, a trend of large immune heterogeneity based on anatomy and histomorphology exists within subtypes.
