# Supplementary material for: Enhanced measures of neoantigenicity capture unique tumor-immune interactions across primary melanoma subtypes
Source: Genome Med. 2026 May 26;18:106. doi: 10.1186/s13073-026-01673-3 (PMC13390141; doi:10.1186/s13073-026-01673-3)
Supplement: Supplementary file 4 — Supplementary Material 4. [file 13073_2026_1673_MOESM4_ESM.pdf]

Supplementary Methods

Copy number profiles, purity and ploidy. Inspection of purity against ploidy values revealed a bias of ascatNgs purity estimates where tumors with higher ploidy had higher purity values. In addition, ascatNgs estimates a purity of 100% in some samples, unrealistic for FFPE-derived DNA and in high disagreement with Sequenza purity estimates. In contrast, Sequenza did not show an association with ploidy estimates and did not assign 100% purity values. Hence, the Sequenza purity estimates were used for downstream analysis. For ploidy, those tumors with a ploidy difference less than 0.1 between both methods were regarded as in agreement. This threshold allows for no more than a difference of two chromosome level amplifications or deletions in the overall ploidy estimation. For those tumors that disagree, the following steps were taken: (i) inspection of percentage of bi-allelic loss (BAL) revealed that Sequenza would overcall BAL, hence for those samples with a percentage BAL >4% in Sequenza but <=4% in ascatNgs, the ascatNgs ploidy solution was chosen. No samples with a BAL >4% in ascatNgs but <=4% in Sequenza were identified. (ii) for samples with an estimated ploidy greater than 4 by one of the methods, we inspected the Mitelman database (https://mitelmandatabase.isb-cgc.org, accessed 26 October 2020) to explore how prevalent such high-ploidy melanomas were present. In Mitelman, 2.6% (3/114) of melanomas were identified to have a ploidy >4, whereas Sequenza and ascatNgs identified 11.7% (14/119) and 5.8% (7/119) of melanomas to have ploidy>4, respectively. Hence, if a sample had a ploidy estimate>4 according to Sequenza and a ploidy estimate<=4 according to ascatNgs, the latter solution was used. The same rationale applied for samples that had a ploidy estimate>4 according to ascatNgs and a ploidy estimate<=4 according to Sequenza. (iii) Inspection of samples with a ploidy <=2.5 by one method and ploidy >2.5 and <4 by the other method (with an overall disagreement equal or greater than 0.1) indicated disagreement in the basal ploidy level of the sample likely due to a potential whole-genome duplication (WGD) event. To tackle this disagreement, the following approach was applied: First, the extent of allelic balance (AB) in autosomal regions (the percentage of the segmented autosomal genome that presents the same number of minor and major alleles) was calculated for samples that agreed in ploidy estimate and in WGD status (estimated as previously published [1]) between both methods. Second, the AB distribution between samples with no WGD and with WGD for samples that agree in ploidy estimate between both methods was compared with a Wilcoxon test, yielding an expected highly significant p-value (p-value<10-40) given the known higher levels of allelic imbalance in tumors with WGD events [1]. Third, this set of samples was used as a training set to generate a

logistic regression model using the glm function in R (v3.6.2), using WGD status as the response variable and the extent of AB as the regressor. To ensure robustness of the model, this component of the methodology was done on the superset of samples from the Australian Melanoma Genome Project (AMGP) [2]. Fourth, for samples that disagree in ploidy (ploidy <=2.5 by one method and ploidy >2.5 and <4 by the other method), the trained logistic regression model and the predict function with type=response was used to calculate the probability of a WGD event given each samples' extent of AB. If both methods (sequenza and ascatNgs) yielded a probability >0.5 of WGD, the higher ploidy estimate was used. If both methods (Sequenza and ascatNgs) yielded a probability <=0.5 of WGD, the lower ploidy estimate was used. If a method yielded a probability <=0.5 (indicative of no WGD) and the other method a probability>0.5 (indicative of WGD), then the solution with a probability value furthest from 0.5 (denoting a higher degree of certainty in the call) was used. (iv) Finally, For the remaining samples that had disagreement in ploidy estimate, a finite mixture model with k=2 and gamma distribution was used with the *flexmix* function from the flexmix package v2.3-17 on the ploidy estimates of samples that agreed between methods using the superset of AMGP cohort for robustness of the model. Based on the shape and the rate estimates of each cluster, the mean (shape/rate) and standard deviation (shape/rate2) were calculated for each group. The clusters had a mean of 1.9 and 3.4, respectively, indicative of the expected bimodal ploidy distribution of the cohort. Then, the ploidy estimate generated by a method (Sequenza or ascatNgs) closest to either ploidy mean was chosen as the solution. The allele-specific copy number profile utilised for each sample corresponds to the one generated by the method for which the ploidy estimate was chosen with the procedure described above.

References

1. Vergara, I. A. *et al.* Evolution of late-stage metastatic melanoma is dominated by aneuploidy and whole genome doubling. *Nat Commun* **12**, 1434 (2021). https://doi.org:10.1038/s41467-021-21576-8
2. Newell, F. *et al.* Comparative Genomics Provides Etiologic and Biological Insight into Melanoma Subtypes. *Cancer Discov* **12**, 2856-2879 (2022). https://doi.org:10.1158/2159-8290.CD-22-0603
